# Zinc Supplementation Enhances the Pro-Death Function of UPR in Lymphoma Cells Exposed to Radiation

**DOI:** 10.3390/biology11010132

**Published:** 2022-01-13

**Authors:** Roberta Gonnella, Luisa Guttieri, Maria Saveria Gilardini Montani, Roberta Santarelli, Erica Bassetti, Gabriella D’Orazi, Mara Cirone

**Affiliations:** 1 Department of Experimental Medicine, “Sapienza” University of Rome, 00161 Rome, Italy; roberta.gonnella@uniroma1.it (R.G.); mariasaveria.gilardinimontani@uniroma1.it (M.S.G.M.); roberta.santarelli@uniroma1.it (R.S.); 2 Laboratory Affiliated to Istituto Pasteur Italia-Fondazione Cenci Bolognetti, 00161 Rome, Italy; 3 Department of Clinical and Molecular Medicine, Sapienza University of Rome, 00161 Rome, Italy; luisa.guttieri@uniroma1.it; 4 Department of Radiological, Oncological and Pathological Sciences, “Sapienza” University of Rome, 00161 Rome, Italy; e.bassetti@policlinicoumberto1.it; 5 Department of Neurosciences, Imaging and Clinical Sciences, University “G. D’Annunzio” Chieti, 66013 Chieti, Italy; gdorazi@unich.it; 6 Department of Research and Technological Innovation, IRCCS Regina Elena National Cancer Institute, 00161 Rome, Italy

**Keywords:** ER stress/UPR, DDR, CHOP, ERK1/2, p53, PEL

## Abstract

**Simple Summary:**

It is of fundamental importance to find strategies able to reduce the minimum doses of anticancer treatments, such as radiations, and concomitantly maintain efficient killing of cancer cells. The interconnection between ER stress and DNA repair may represent a promising approach to obtaining this goal. Here we found that pretreatment of lymphoma cells with Zinc chloride rendered these cells more sensitive to 2 Gy radiation. The exacerbation of ER stress and the activation pro-death function of UPR were among the underlying mechanisms leading to higher cytotoxicity of Zinc/radiation combination treatment. This evidence encourages the use of Zinc to reduce the doses of radiation in the treatment of lymphoma cells, allowing a high cytotoxicity to be obtained while minimizing the side effects.

**Abstract:**

We have previously shown that Zinc supplementation triggered ER stress/UPR in cancer cells undergoing treatment by genotoxic agents, reactivated wtp53 in cancer cells harboring mutant p53 (mutp53) and potentiated the activity of wtp53 in those carrying wtp53. In this study, we used Zinc chloride alone or in combination with 2 Gy radiation to treat Primary Effusion Lymphoma (PEL) cells, an aggressive B-cell lymphoma associated with KSHV that harbors wt or partially functioning p53. We found that Zinc triggered a mild ER stress/UPR in these lymphoma cells and activated ERK1/2, molecule known to sustain cell survival in the course of UPR activation. In combination with radiations, Zinc triggered a stronger p53 activation that counteracted its mediated ERK1/2 phosphorylation, further upregulating the UPR molecule CHOP and promoting cell death. These data suggest that Zinc supplementation could be a promising strategy to reduce the doses of radiation and possibly of other DNA-damaging agents to obtain an efficient capacity to induce lymphoma cell death.

## 1. Introduction

ZnCl2 (hereafter Zinc) has been previously demonstrated to regulate the biology of p53, as it can reactivate mutant p53 (mutp53), restoring the chemosensitivity to anticancer treatments of cancer cells carrying such mutation [1,2]. We later demonstrated that Zinc, besides reactivating mutp53, triggered Endoplasmic Reticulum (ER) stress and Unfolded Protein Response (UPR) in cisplatin and adriamycin (ADR)-treated mutp53 colon cancer cells, leading to an immunogenic cell death (ICD) [3]. Cisplatin and ADR are genotoxic drugs that, as such, induce DNA damage and trigger the DNA damage response (DDR), which may reduce the efficacy of such treatments. DDR comprises several DNA repair pathways that go from the base and nucleotide excision repair (BER and NER), activated in response to DNA single-strand breaks, to the Non-Homologous End Joining (NHEJ) repair and Homologous Repair (HR) pathways activated by DNA double strand breaks. HR comprises several molecules essential for the execution of DNA repair, including RAD51 and BRCA-1. Regarding wtp53, Zinc has been shown to contribute to its activation, promoting cell death in colon cancer cells undergoing low-dose Adriamycin (ADR) that, as single treatment, was not able to induce such effect [4].

As DDR, UPR is a protective response that helps cancer cells to survive, particularly in the course of anticancer treatments. However, in conditions of too-strong or prolonged stress, it may promote cell death, mainly through the upregulation of C/EBP homologous protein (CHOP) [5]. Interestingly, UPR and DDR, mainly activated by proteotoxic and genotoxic stress, respectively, communicate and share several endpoint molecules that dictate the final outcome in terms of cell death/survival decision [6]. p53 and ERK1/2 are among the molecules that, being regulated by both UPR and DDR activation, may orchestrate the crosstalk between the two responses. Indeed, besides DNA damage [7], UPR activation has been reported to transcriptionally upregulate p53 [8] or downregulate it during ER stress [9]. ER stress/UPR may promote ERK1/2 phosphorylation, mainly through the Ire1 alpha arm, which prevents the apoptotic function of UPR [10], for example by positively regulating the antiapoptotic members of BCL2 family proteins [11]. Interestingly, ERK1/2 may also be activated by DNA-damaging agents and prevent cell death in cancer cells undergoing such treatments [12,13]. p53 and ERK1/2 usually play an opposite role in cancer cell survival, as p53 activation may induce cell cycle arrest or apoptosis [14] while ERK1/2, a pathway activated downstream of growth factor receptors or RAF/RAF protein kinases, essentially sustains cancer cell growth [15]. However, depending on the duration of its activation and the cellular contexts, ERK1/2 can also promote cell death, possibly in correlation with its impact on autophagy [16].

An important aspect to consider is the interplay that occurs between ERK1/2 and p53 [17], as ERK1/2 may phosphorylate p53 [18] and p53 may dephosphorylate ERK1/2 [19]. For example, it can upregulate dual-specificity phosphatase (DUSP) 5 [20] or Raf kinase inhibitor RKIP [21]. Based on this background, in this study, we investigated how ERK1/2 and p53 could affect DNA damage UPR and cell death/cell survival decision in lymphoma cells treated by Zinc and exposed to 2 Gy radiation. We used cell lines derived from Primary Effusion Lymphoma (PEL), a malignant lymphoma associated with KSHV and characterized by an aggressive behavior and a poor response to conventional anticancer therapies [22]. New and more effective treatments for this lymphoma are needed.

## 2. Materials and Methods

### 2.1. Cell Cultures and Treatments

BC3 (ATCC, CRL-2277) and BCBL1 are human B-cell lines derived from PEL (Primary Effusion Lymphomas) which harbor KSHV virus in a latent state. BC3 cell line was purchased from ATCC (CRL-2277) and BCBL1 was kindly supplied by Prof. P. Monini (National AIDS center, Istituto Superiore di Sanità, Rome, Italy). Cells were cultured at 37 °C in a 5% CO_2_ humified setting in the presence of RPMI-1640 medium (Sigma-Aldrich, St. Louis, MO, USA, R0883) supplemented with: 10% Fetal Bovine serum (FBS Euroclone, Milano, Italy, ECLS0180L), antibiotic mixture containing streptomycin (100 µg/mL) and penicillin (100 U/mL) and l-glutamine (2 mM) (GIBCO Gaithersburg, MD, USA, 10378-016). Cells were seeded in 6-well plates at a density of 2.5 × 10^5^/mL and treated with Pifithrin-α (p53 inhibitor Sigma-Aldrich CAS 506132), PD98059 (ERK inhibitor Sigma-Aldrich P215) and ZnCl_2_ (Sigma-Aldrich cat n. 208086) at 20, 10, 25 and 50 μM, respectively. Cells were incubated with drugs for 5 h then they were irradiated with 2 Gray (2 Gy) radiation and 24 h later they were collected in order to assess cell viability and extract proteins.

### 2.2. Cell Viability

Cell viability was determined by Trypan Blue (Sigma-Aldrich, St. Louis, MO, USA, R0883) exclusion assay after 24 h of culture, as already described [23].

### 2.3. Apoptosis Analysis

ANXA5 staining was performed using the Annexin V-FITC Apoptosis Detection Kit (PharMingen, San Diego, CA, USA, 556,547). After treatments, cells were washed and incubated with 5 μL of AV-FITC for 15 min at room temperature in the dark, followed by addition of 400 μL of 1× binding buffer. Samples were analyzed with a FACSCalibur flow cytometer, using CELLQuest software (BD Biosciences, San Jose, CA, USA).

### 2.4. siRNA Interference

For p53 silencing 2.5 × 10^5^ cells (BC3 and BCBL1) were seeded in six-wells culture plates, empty vector (Ref) and Si-p53 plasmid (specific for p53 interference) were subsequently used for transfection by using Lipofectamin 3000 (Invitrogen, Life Techmologies, Carlsbard, CA, USA). For each well, 5 µg of plasmid DNA were used and 24 h later cells were treated with ZnCl_2_ for 1 h then irradiated with 2 Gy. The day after, the cells viability was assessed by trypan blue exclusion assay and finally cells were harvested for further experiments.

### 2.5. Western Blot Analysis

After treatments, cells were collected, centrifuged and finally lysed in modified RIPA buffer and analyzed as already described [23]. All original western blots are reported in Figure S1.

### 2.6. Antibodies

The following primary antibodies were used for Western blot analysis: mouse monoclonal anti-ATF4 (NovusBIO, CO, USA, cat# MAB7218) 1:3000, rabbit polyclonal anti-BiP/GRIP78 (Proteintech, Rosemont, IL, USA, cat# 11587-1-AP) 1:5000, rabbit polyclonal anti-CHOP (GADD153) (Proteintech, cat# 15204-1-AP) 1:1000, mouse monoclonal anti-DUSP5 (Santa Cruz Biotechnology Inc., Dallas, TX, USA cat# sc-393801) 1:200, rabbit polyclonal anti-ERK1 (Santa Cruz Biotechnology Inc. cat# sc-93) 1:200, rabbit polyclonal anti-ERK2 (Santa Cruz Biotechnology Inc. cat# sc-154) 1:200, mouse monoclonal anti-p-ERK (Santa Cruz Biotechnology Inc. cat# sc-7383) 1:500, mouse monoclonal *anti*-pH2AX (Ser 139) (Santa Cruz Biotechnology Inc. cat# sc-517348) 1:100, rabbit polyclonal anti-PARP1 (Proteintech, cat# 13371-1) 1:1000, rabbit polyclonal *anti*-p21 (clone C-19, Santa Cruz Biotechnology Inc. cat# sc-397) 1:200, mouse monoclonal *anti*-p53 (clone DO-1, Santa Cruz Biotechnology Inc. cat# sc-126) 1:100 and Mouse monoclonal *anti*-β-actin (Sigma Aldrich cat# A5441) 1:10,000 was used as loading control. Goat anti-rabbit IgG-horseradish peroxidase HRP (Santa Cruz Biotechnology, Inc. Heidelberg, Germany, cat# sc-2004) 1:10,000, goat anti-mouse IgG-horseradish peroxidase HRP (Santa Cruz Biotechnology, Inc. Heidelberg, Germany, cat#sc-2005) 1:10,000 were used as secondary antibodies. All primary and secondary antibodies used in this study were diluted in a PBS 0.2% Tween 20 solution containing 3% BSA.

### 2.7. Densitometric Analysis

Densitometric analysis of Western blot bands was performed by using the ImageJ software, which was downloaded from the NIH web site (http://imagej.nih.gov; accessed on 23 November 2021, version 1.41o, NIH, Bathesda, MA, USA).

## 3. Results

### 3.1. Zinc Triggers Mild ER Stress, Increases ERK1/2 Phosphorylation and Slightly Affects Cell Survival in PEL Cells

**Figure 1:**
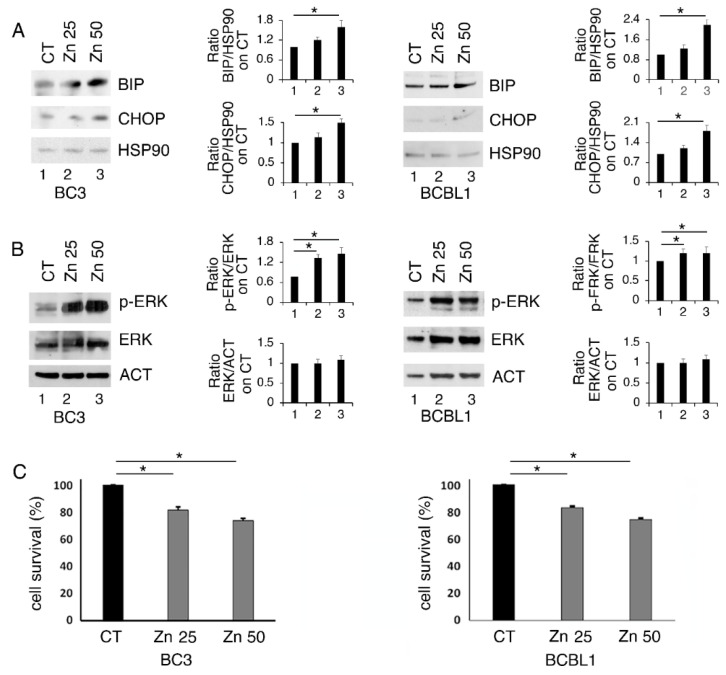
Zinc upregulates BiP and slightly CHOP and increases ERK1/2 phosphorylation in BC3 and BCBL1 cells. BC3 and BCBL1 were untreated (CT) and treated with 25 and 50 μM Zinc (Zn) and the expression level of BiP and CHOP (**A**) and phospho- and total ER1/2 (**B**) were evaluated by Western blot analysis. Densitometric analysis was performed as reported in material and methods. (**C**) BC3 and BCBL1 cells were treated with 25 and 50 μM Zinc (Zn) and cell survival was evaluated by trypan blue assay after 24 h of treatment, % of cell survival is shown. The histograms represent the mean plus S.D. from three experiments. * *p*-value < 0.05.

BC3 and BCBL-1 PEL cells, harboring wt and partially functioning p53 [24], were treated with 25 and 50 μM of Zinc for 24 h and its impact of on ER stress/UPR, basally activated in these lymphoma cells [23], was evaluated. At this aim, the expression level of BiP and CHOP, which correlates with the pro-survival and pro-death UPR activation, respectively, was investigated. As shown in Figure 1A, BiP and, to a lesser extent, CHOP were upregulated by the highest dose of Zinc, particularly in BC3 cells, suggesting a mild UPR activation. Among the pathways activated in the course of UPR is ERK1/2, a MAPK that has been reported to help cancer cells to face stressful conditions [10]. Here, we found that ERK1/2 phosphorylation increased following Zinc treatment (Figure 1B), an effect that has also been previously reported to be induced by Zinc in other cancer cell types [25]. We then assessed whether Zinc treatment could affect PEL cell survival and found that it was also slightly reduced at the highest dose (Figure 1C).

### 3.2. ERK1/2 Inhibition Upregulates CHOP and Reduces the Survival of Zinc-Treated PEL Cells

**Figure 2:**
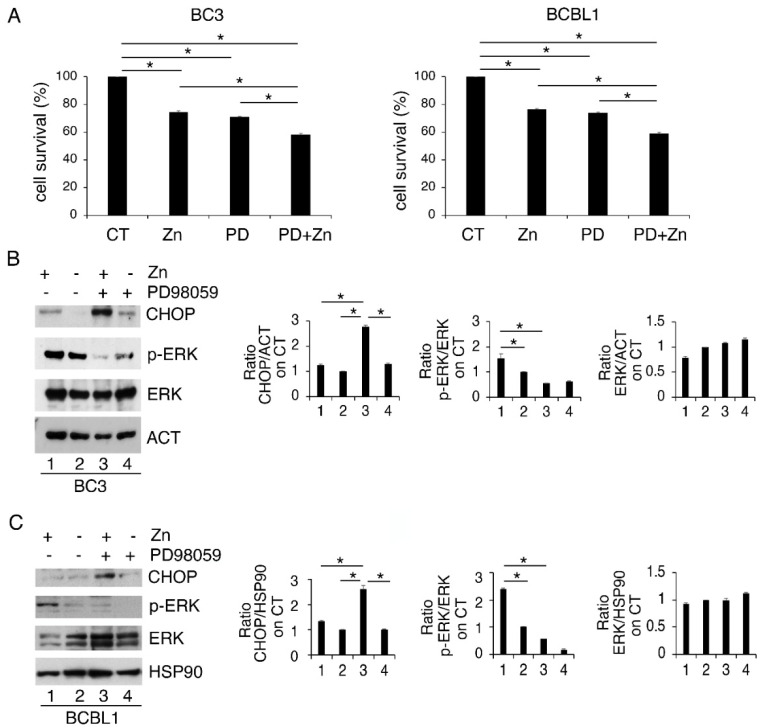
ERK1/2 inhibits CHOP upregulation and sustains cell survival of Zinc-treated cells. (**A**) BC3 and BCBL1 were treated with Zinc in combination or not with PD98059 (PD) and (**A**) cell survival was evaluated by trypan blue assay after 24 h of treatment; (**B**,**C**) CHOP expression level and phospho- and total ERK1/2 were analyzed by Western blot. Densitometric analysis was performed as reported in Materials and Methods. Histograms represent the mean plus SD of three different experiments. * *p*-value < 0.05.

We then evaluated the role of ERK1/2 phosphorylation, induced by Zinc, on PEL cell survival. With this aim, we inhibited it by PD98059 (PD) before Zinc treatment and found that such combination further impaired cell survival compared to the single treatments (Figure 2A). In correlation with the reduction in cell survival, ERK1/2 dephosphorylation led to a higher expression level of the pro-mortem UPR molecule CHOP (Figure 2B). These results indicate that ERK1/2 activation plays a pro-survival role in Zinc-treated cells, preventing CHOP upregulation and the pro-death function of UPR. In agreement with this finding, previous studies have shown a pro-survival role of ERK1/2 cells in the course of ER stress correlated with its mediated CHOP downregulation [26].

### 3.3. Zinc in Combination with Low-Dose Radiation Enhances DNA Damage and p53 Activation, Reduces ERK1/2 Phosphorylation, Upregulates ATF4/CHOP Axis and Further Impairs PEL Cell Survival

**Figure 3:**
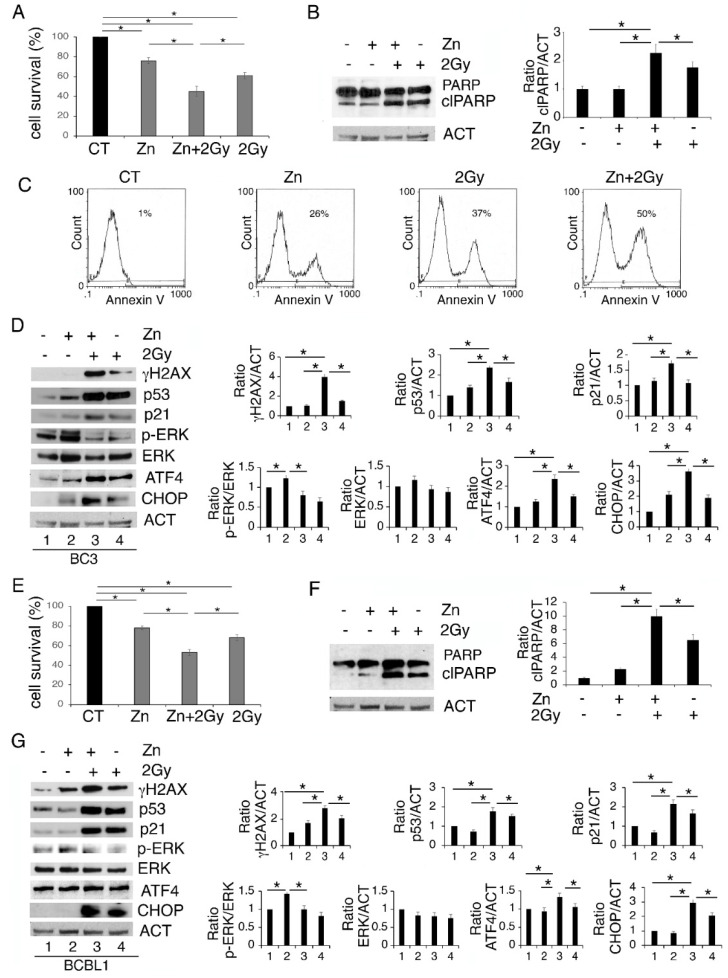
Zinc enhances DNA damage and p53 activation induced by radiation, reduces ERK1/2 phosphorylation, upregulating ATF4/CHOP axis and further impairing PEL cell survival. BC3 and BCBL1 were treated with Zinc and then exposed to 2 Gray (Gy) radiation. After 24 h of treatment cell survival was evaluated by trypan blue assay (**A**,**E**) and the expression level of PARP1 (**B**,**F**), *γ*H2AX, p53, p21, phospho- and total ERK1/2, ATF4 and CHOP were analyzed by Western blot (**D**,**G**). Densitometric analysis was performed as reported in Material Methods. Histograms represent the mean plus SD of three different experiments. (**C**) FACS profiles showing AnnexinV staining of BC3 cells undergoing the indicated treatments. * *p*-value < 0.05.

We then investigated whether Zinc could enhance PEL cell death induced by 2 Gy radiation. As shown in Figure 3A,E, Zinc increased the cytotoxic effect of low-dose radiation both in BC3 and BCBL-1 cells. Accordingly, PARP cleavage (clPARP) increased in cells undergoing Zinc/radiation combination treatment (Figure 3B,F), which also suggests the occurrence of apoptotic cell death. The apoptotic effect was confirmed by annexin V staining that increased in BC3 cells, particularly following Zinc/radiation treatment (Figure 3C). ER stress/UPR activation have been shown to influence DNA repair following treatment with genotoxic agents [27]; therefore, next we investigated whether Zinc-enhanced cell death could correlate with increased DNA damage in cells exposed to low-dose radiation. At this aim we assessed the appearance of the phosphorylated form of H2AX (*γ*H2AX) and, as shown in Figure 3D,G, we found that radiation increased *γ*H2AX and that it further increased with Zinc/radiation combination. The expression level of p53, classically activated by DNA damage, was then investigated. In correlation with *γ*H2AX, a stronger activation of p53 and its target p21 were observed in cells undergoing a Zinc/radiation combination compared to the single-radiation treatment (Figure 3D,G). This suggests that Zinc could promote PEL cell death by enhancing DNA damage and contributing to p53 activation induced by radiation. Given that ERK1/2 was activated by Zinc in PEL cells and given that crosstalk between p53 and ERK1/2 has been previously reported [17], we then evaluated the status of ERK1/2 phosphorylation in PEL cells treated with Zinc and exposed or not to radiation. As shown in Figure 3D,G, concomitantly with p53 activation, ERK1/2 phosphorylation was reduced by the combined treatment. We also found that the expression of the transcription factor ATF4, activated during UPR, and the CHOP molecule mainly upregulated downstream of it further increased following the combined treatment. As the pro-death function of UPR mainly correlates with CHOP upregulation, this could contribute to the higher cytotoxic effect exerted by Zinc in combination with radiation.

### 3.4. p53 Activation Contributes to ERK1/2 Dephosphorylation in PEL Cells Undergoing Zinc/Radiation Treatment

**Figure 4:**
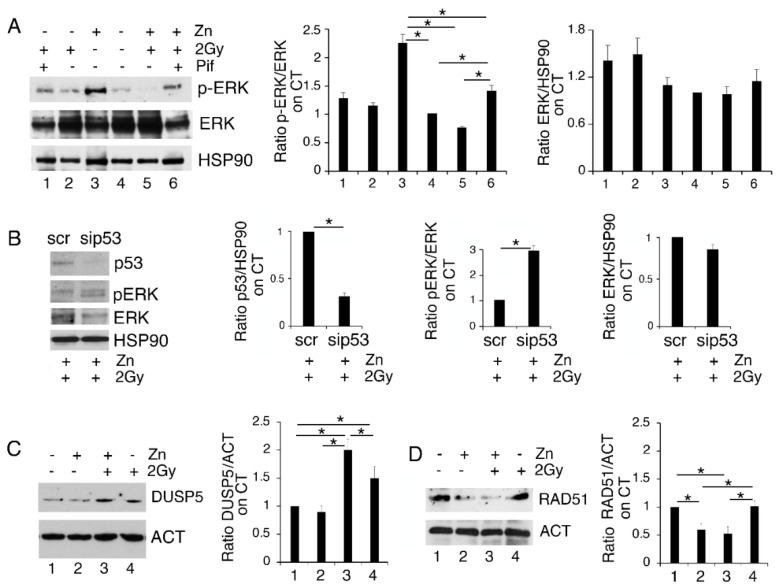
The activation of p53 is involved in ERK1/2 dephosphorylation in PEL cells treated with Zinc/radiation. (**A**) BC3 cells were treated with Zinc/2Gy radiation in the presence or in the absence of pifithrin-*α* (pif) and phospho- and total ERK1/2 were assessed by Western blot analysis; (**B**) BC3 cell were silenced for p53 and the expression of p53, phospho and total ERK1/2 were analyzed by Western blot; (**C**,**D**) BC3 cells treated with Zinc in combination or not with 2Gy radiation were analyzed in Western blot for the expression of DUSP5 or RAD51. Actin or HSP90 were used as loading control. Densitometric analysis was performed as reported in Material Methods. Histograms represent the mean plus SD of three different experiments, * *p*-value < 0.05.

To investigate whether p53 activation could play a role in reducing ERK1/2 phosphorylation in Zinc/radiation-treated PEL cells, we inhibited p53 activity by using Pifithrin-*α* (Pif). As shown in Figure 4A, this drug was able to partially restore ERK1/2 phosphorylation in PEL cells treated with a Zinc/radiation combination, suggesting involvement of p53 in ERK1/2 dephosphorylation. This was confirmed by the silencing of p53 in BC3 treated with Zinc/radiation that showed and increase in ERK1/2 phosphorylation in comparison to the scramble-treated cells (Figure 4B). Several phosphatases such as the Dual-Specificity Phosphatase (DUSP) 5, reported to be transcriptionally upregulated by p53, have been shown to target ERK1/2 [20]. Here we found that DUSP5 was upregulated in PEL cells exposed to radiation and Zinc/radiation-combination treatments (Figure 4C), which suggests that this phosphatase could play a role in ERK1/2 inhibition in PEL cells undergoing this treatment.

Both p53 activation [24,28] and ERK1/2 dephosphorylation [29] negatively regulate DNA repair by reducing the expression level of RAD51. This molecule, essential to the HR pathway, has also been previously reported to be degraded in the course of ER stress [30]. Therefore, we then investigated the expression level of this molecule and, as shown in Figure 4D, Zinc treatment also reduced RAD51 expression in combination with radiation. Therefore, the reduction in DNA repair in cells exposed to DNA damage by low-dose radiation could be one of the reasons why Zinc exacerbates DNA damage, stress and cell death when combined with radiation.

## 4. Discussion

**Figure 5:**
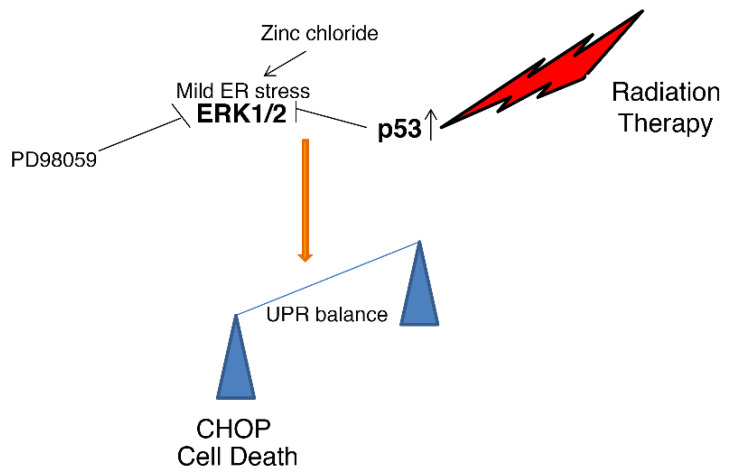
Representative scheme illustrating how the interaction between wtp53 and ERK leads to UPR umbalance and cell death.

This study evidenced that Zinc-induced ER stress and ERK1/2 activation enhanced cell death in PEL cells exposed to radiation. At the molecular level, we found that radiation activated p53, that it reduced ERK1/2 phosphorylation induced by Zinc, and that such a combination increased DNA damage and further activated p53, upregulating the pro-mortem UPR molecule CHOP (Figure 5). The protective role of ERK1/2 activated by Zinc treatment was evidenced in this study by the use of PD98059, which led to CHOP upregulation and increased PEL cell death. Accordingly, ERK1/2 activation by ER stress has been previously shown to downregulate CHOP or upregulate BiP in other cancer cell types, to sustain cell survival [11]. Conversely, ERK1/2 inhibition downregulates the pro-survival UPR molecule BiP (GRP78) and skews the balance of UPR towards cell death [31].

Interestingly, ERK1/2 has also been reported to play a role in the prevention of cell death in cancer cells exposed to radiation [32], treatment that classically induces DNA damage and activates p53. ERK1/2 and p53 usually play opposite roles in the cell death/survival decision of cells undergoing anticancer treatments and, interestingly, p53 may inhibit ERK1/2 activation, i.e., by inducing the transcription of phosphatases such as DUSP5 [20]. Accordingly, here we found that concomitantly to p53 activation and ERK1/2 dephosphorylation, DUSP5 was activated by Zinc/radiation treatment. This study contributes to highlighting the emerging finding that UPR and DDR are strongly inter-connected processes and that molecules such as ERK1/2 and p53 can act as a bridge between these responses [6,32]. It was previously reported that molecules belonging to the DNA repair pathways, such as RAD51 or molecules belonging to UPR, such as Ire1alpha, can be activated by both ER stress and DDR signaling [6,30,33]. Moreover, UPR and DDR share several biological endpoints molecules that ultimately dictate cell fate, sustaining cell survival, when the cell damage is bearable, while promoting cell death when cells cannot further recover from it [6]. Exploring the molecules that lie at the intersection between UPR and DDR is particularly important as their concomitant targeting may offer new therapeutic opportunities in cancer treatment. Indeed, besides inhibitors of DNA repair molecules, drugs affecting UPR sensors can be exploited to improve the cytotoxic effect of DNA-damaging agents. On the other hand, it could be possible to exacerbate the cytotoxicity of drugs inducing ER stress by also inhibiting DNA repair molecules in combination with UPR sensors’ inhibition.

## 5. Conclusions

In conclusion, this study suggests that the inhibition of ERK1/2, activated by Zinc in the course of its induced ER stress, exacerbates the pro-death function of UPR in lymphoma cells. The activation wtp53 by DNA damage following exposure to radiation also counteracts ERK1/2 phosphorylation in Zinc-treated cells and exacerbates stress and DNA damage, further impairing lymphoma cell survival.

## Data Availability

The datasets generated and/or analyzed during the current study are available from the corresponding author upon reasonable request.

## Supplementary Materials

The following supporting information can be downloaded at: https://www.mdpi.com/article/10.3390/biology11010132/s1, supplementary figure containing original western blots.
