# Supplementary figures and images for: Zinc Supplementation Enhances the Pro-Death Function of UPR in Lymphoma Cells Exposed to Radiation
Source: Biology (Basel). 2022 Jan 13;11(1):132. doi: 10.3390/biology11010132 (PMC8773084; doi:10.3390/biology11010132)

Figure S1

A

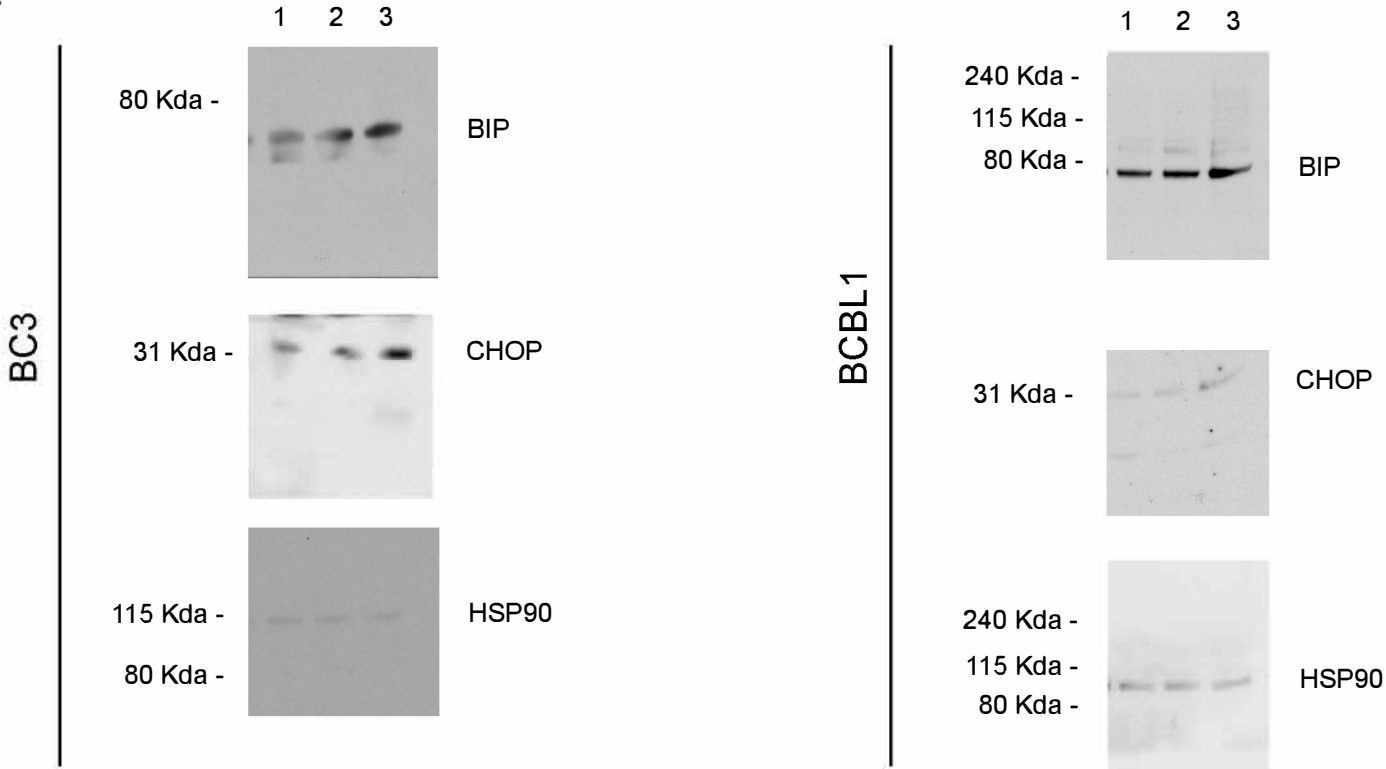

B

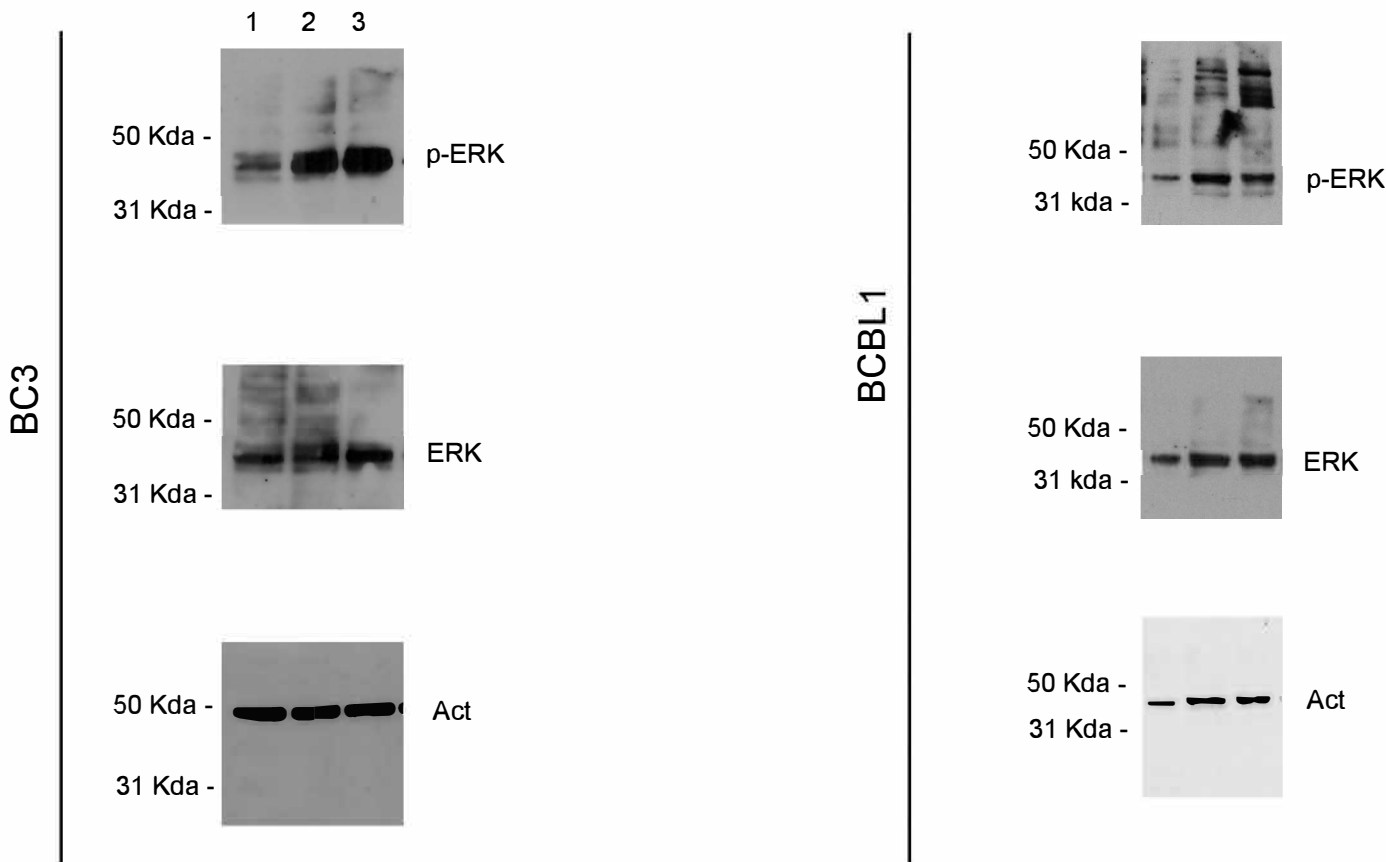

Figure S2

B

BC3

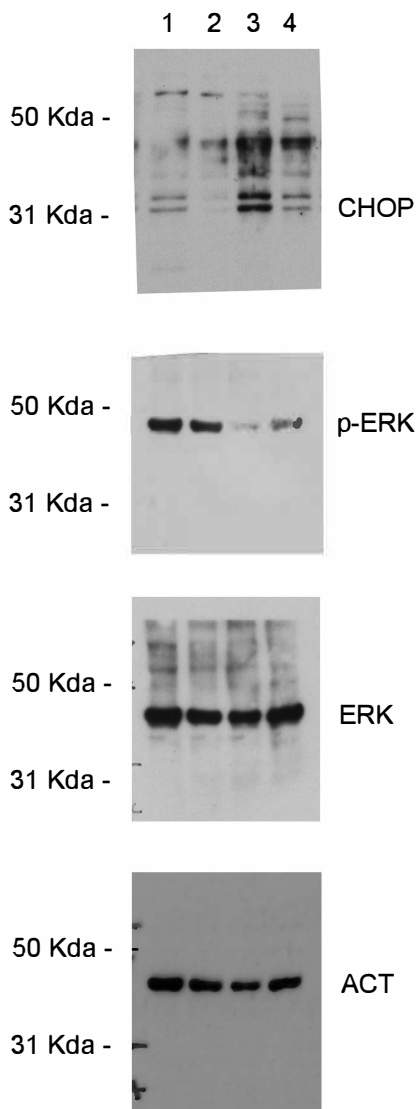

C

BCBL1

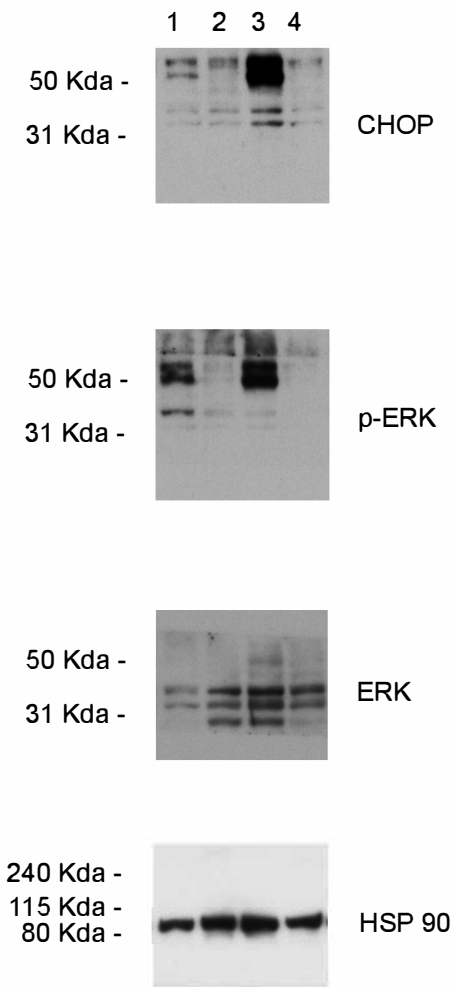

Figure S3

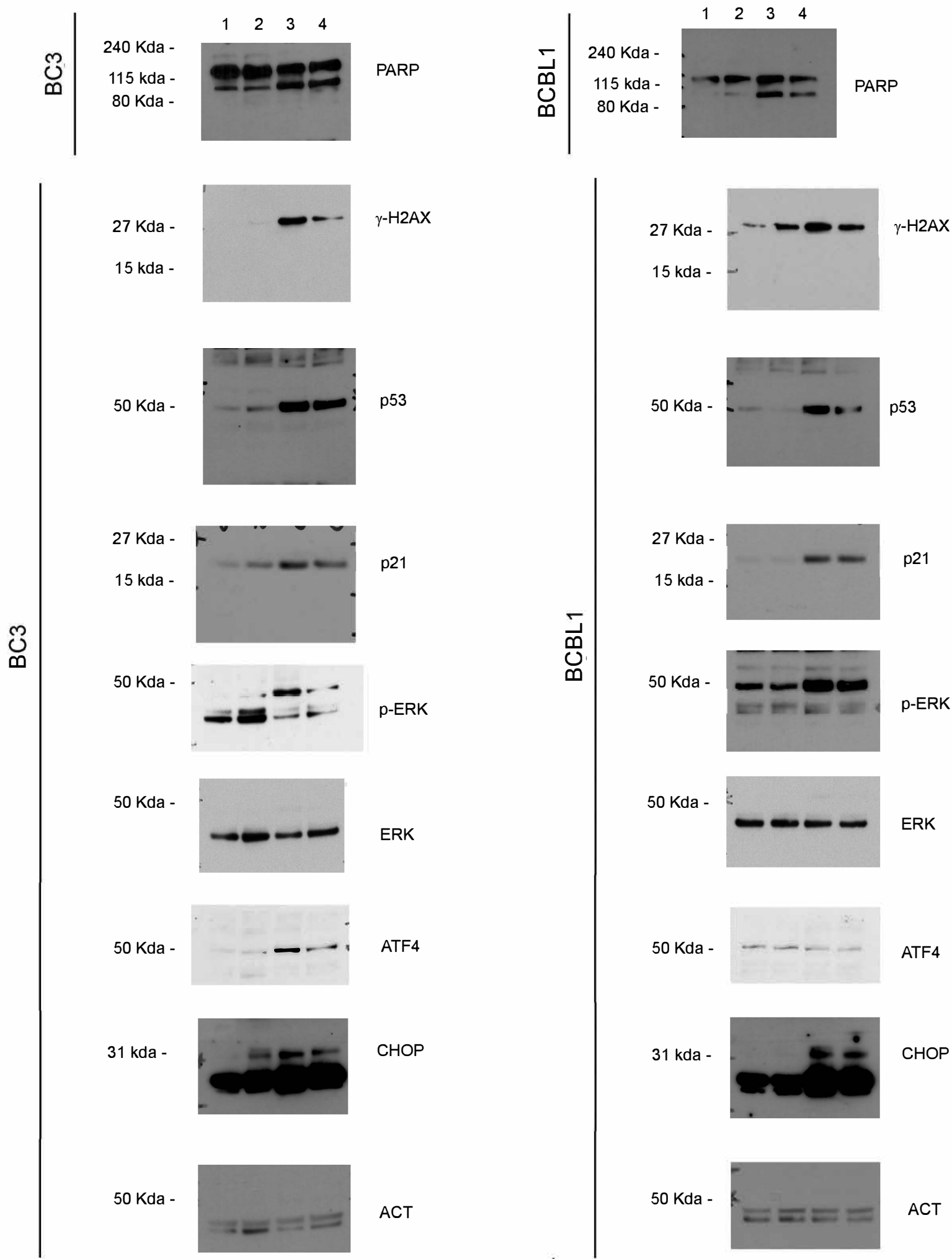

Figure S4

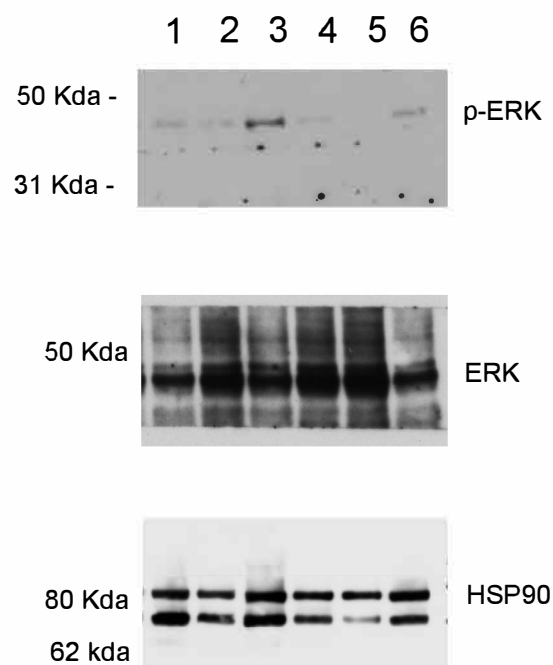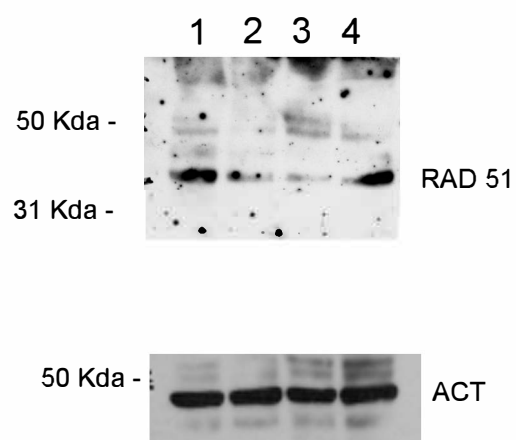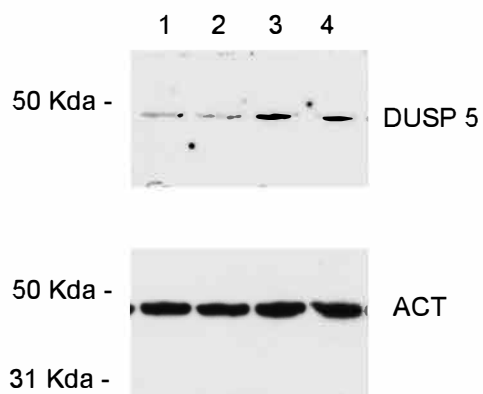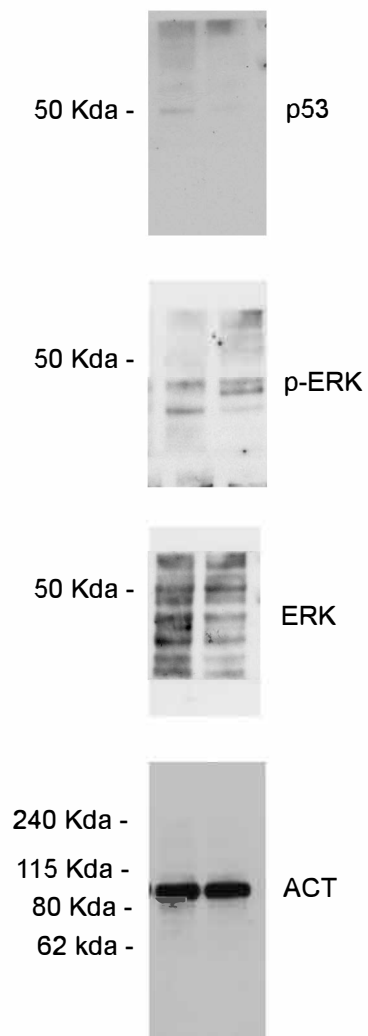

Supplement: Supplementary file 1 [file biology-11-00132-s001.zip › biology-1501847-supplementary.pdf]
